# A self-assembled Dendrobium polysaccharide hydrogel with anti-aging activity via the FOXO signaling pathway

**DOI:** 10.1007/s13659-026-00642-y

**Published:** 2026-10-01

**Authors:** Yanye Zhang, Yuanhe Wang, Huiyan Shao, Xingli Guo, Pengcheng Xu, Yanlang Li, Jianke Jia, Fawu Dong, Jiang-miao Hu, Liu Yang

**Affiliations:** 1 Yunnan Key Laboratory of Chinese Medicine Processing, College of Chinese Materia Medica, Yunnan University of Chinese Medicine, Kunming, 650500 China; 2 Key Laboratory of Phytochemistry and Natural Medicines, and Key Laboratory of Phytochemistry and Plant Resources in West China, Kunming Institute of Botany, Chinese Academy of Sciences, Kunming, 650201 Yunnan China

**Keywords:** Dendrobium polysaccharide composite hydrogel, Anti-aging, FOXO signaling pathway

## Abstract

Faced with the complexity and diversity of skin aging, it is critical to investigate multi-target synergistic anti-aging products. Herein, a novel composite hydrogel with anti-aging activity was developed based on Dendrobium polysaccharide, hyaluronic acid (HA), and nicotinamide (NMN). The hydrogen bond and van der Waals force were the main driving forces in the self-assembly interaction between Dendrobium polysaccharides (DDAM-1 and DOMOS), HA, and NMN. They also were important molecular mechanisms for the gel formation of the composite hydrogel, and the results have been confirmed by ITC and molecular docking. The composite hydrogel demonstrated potent anti-aging effect on the skin in vivo and in vitro, including inhibited elastase/collagenase, promoted type I collagen secretion, prevented ROS, and reduced the expression of IL-6/MMP-3. In addition, the anti-aging mechanism of the composite hydrogel may be associated with FOXO and TGF-β/Smad- SIRT1 signaling pathways mediated by FOXO3, SIRT1, SMAD3 and TGF-β. Briefly, this study demonstrates the potential of HA-infused composite hydrogels for use as anti-aging cosmetic products due to their synergistic anti-aging strategies for anti-aging applications.

**Supplementary Information:**

The online version contains supplementary material available at https://doi.org/10.1007/s13659-026-00642-y.

## Introduction

Skin aging is a visible expression of systemic aging, while skin aging is also affected by systemic aging [1, 2]. This bidirectional relationship indicates that the mechanisms of skin aging is complex and diverse [1, 3, 4]. Skin aging, driven by both intrinsic and extrinsic factors, compromises skin structure and function. Structurally, it thins the epidermis and degrades the dermal extracellular matrix (ECM), leading to collagen loss and reduced vascularity. Functionally, this manifests as impaired barrier protection, weakened immunity, and slowed wound healing [2, 5–7]. Over the past decade, an updated and general version of the skin aging characteristics has consolidated the knowledge gained in the field of aging, resulting in ten strongly linked biomarkers and mechanisms of skin aging [8]. For example, senescence-associated β-galactosidase (SA-β-Gal), cell cycle regulators, senescence-associated heterochromatin foci (SAHF), Nuclear lamin B1, telomere, ECM, senescence-associated secretory phenotype (SASP), oxidative stress, autophagy, and MAPK signaling pathway are all the biomarkers and mechanisms of skin aging [9–12]. These biomarkers and mechanisms collectively manifest as skin dullness, thinning, wrinkle formation, and loss of elasticity [13] and in severe cases, may predispose to skin disease. Among them, degradation of ECM—hyaluronic acid, collagen and elastin—accompanied by increased activity of elastin and collagenase, and an imbalance in SASP are seen as key characteristics of skin aging [13, 14]. Due to the complexity and diversity of skin aging, a single target approach is no longer sufficient to address the problem. It is critical to develop safe and efficient multi-target drugs or novel drug delivery systems to address the complex mechanisms of skin aging [15].

Current advancements suggested that hydrogels may be a pivotal delivery system for multi-target drug delivery in the field of anti-aging. Porosity, flexibility, biocompatibility, film-forming ability, and excellent biochemical functionality are the basic causation [16, 17]. Particularly, polysaccharide-based hydrogels have been widely used as ECM-mimicking materials in medical dressings [18], suggesting their potential for the treatment of skin aging. However, the selection of a polysaccharide-based carrier is essential. Dendrobium polysaccharide (DP), for instance, have anti-aging properties that have been suggested by ancient books and modern research. Our previous and others' researches have declared that promoting the expression of ECM, antioxidation, moisturizing effect, and suppressing SASP expression is its anti-aging mechanism [16, 19, 20]. Despite these advantages of DP for anti-aging, hydrogels prepared with DP hardly ever stand alone. Since DP have their drawbacks, such as weak mechanical resistance. Furthermore, hyaluronic acid (HA) alone often yields hydrogels with insufficient mechanical strength for effective cell support [20], while nicotinamide (NMN), though capable of delaying fibroblast senescence, can cause skin irritation [21]. Thus, the development of safe and efficient polysaccharide-based carriers is crucial for the construction of composite anti-aging hydrogels for multi-target drug delivery.

So, in this manuscript, the collaborative co assembly strategy has been used to prepare Dendrobium polysaccharide-based hydrogels to fortify their gel ability and anti-aging activity. Firstly, a polysaccharide-based hydrogel was prepared by self-assembly via hydrogen and oxygen atoms on the hydroxyl in Dendrobium polysaccharides (DDAM-1 and DOMOS) and the amide group in NMN, and hydrogen bonding and van der Waals force were the main driving forces in the self-assembly interaction. This polysaccharide-based hydrogel has a stable 3D porous network structure. In addition, NMN self-assembly was further applied to introduce the anti-aging active molecule HA to form a co-assembled composite hydrogel. The comprehensive in vitro and in vivo experiments be aimed to elucidate the counteract skin aging activity and mechanisms of the composite hydrogel. The composite hydrogel regulated the expression of skin aging biomarkers such as ECM, ROS, SASP, and SA-β-Gal, etc. through FOXO and TGF-β/Smad- SIRT1 signaling pathways. The successful construction of co-assembled composite hydrogels provides a novel coping strategy for the complexity of skin aging and new perspectives on the application of traditional Dendrobium orchids.

## Materials and methods

### Materials and chemicals

The human dermal fibroblast (HDFs) cell line was obtained from Kunming Cell Bank of the Chinese Academy of Sciences (Kunming, China). The MTS Cell Proliferation and Cytotoxicity Detection Kit were purchased from Promega (USA). HDFs-specific medium was purchased from Shanghai Fuheng Biotechnology Co., Ltd. (Shanghai, China). Human procollagen I N-terminal propeptide (PINP) was purchased from Elabscience Technology Co., Ltd. (Wuhan, China). Tris–HCl buffer and N-[3-(2-furanoyl)acryloyl]-leucine-glycine-prolinealanine (FALGPA) were purchased from Shanghai Yuanye Biotechnology Co., Ltd. (Shanghai, China). N-succinyl-alanine-alanine-alanine-p-nitroaniline and porcine pancreatic elastase were purchased from Merck Chemical Technology Co., Ltd. (Shanghai, China). The ROS Detection Kit, β-Galactosidase Kit, and Cell Cycle and Apoptosis Kit were purchased from Beyotime Biotechnology (Shanghai, China). C57BL/6J mice were purchased from the Animal Research and Resource Center of Yunnan University (Kunming, China). D-Galactose was purchased from Sigma-Aldrich (USA). Niacinamide (NMN, CAS:98-92-0) and hyaluronic acid (HA, molecular weight: 3000 Da, CAS:9004-61-9) were obtained from Yunnan Xili Biotechnology Co., Ltd. (Yunnan, China). Antibodies against SIRT1, SMAD3, FOXO3, TGFβ, and GAPDH were sourced from Yunnan Zehao Trading Co., Ltd. (Yunnan, China). Pyridine, 2- [5- (2,4-dichlorophenyl) −1,2,4-oxadiazol-3-yl] - (9CI, ACI) (JY-2) was prepared from Med Chem Express (CAS No. 339103-05-8, molecular weight 292.12 Da). The *Dendrobium officinale* polysaccharide (DOMOS) and *Dendrobium devonianum* polysaccharide (DDAM-1) used in this study were prepared and characterized as previously described [22, 23]. Briefly, DOMOS is a manno-oligosaccharide with a molecular weight of 828 Da and a backbone composed of five monosaccharide residues (three mannose and two glucose). DDAM-1 is an O‑acetyl‑mannan with a molecular weight of 3.89 × 10^4^ Da. Both polysaccharides have been produced at kilogram scale with confirmed batch‑to‑batch consistency, the structural details see in Additional file 1: Fig. S1.

### Cell culture and viability assay

HDFs are routinely cultured with 10% fetal bovine serum and 1% penicillin–streptomycin solution and maintained at 37 °C in a humidified 5% CO_2_ incubator.

HDFs were seeded in 96-well plates and cultured for 24 h. After discarding the supernatant, the cells were treated with pre-diluted test compounds, with a blank control group established as reference. Each treatment group consisted of five replicates and was incubated at 37 °C. At the designated time points (1, 24, and 48 h), the culture medium was removed. Absorbance was measured by performing the MTS assay in line with the provided procedure. The cellular survival rate, expressed as RC, was then calculated using formula (1).

1 $$RC\left( \% \right) = \left( {A_{s} - A_{b} } \right)/\left( {A_{c} - A_{b} } \right) \times 100\%$$

In the formula, A_s_ (Sample): Absorbance values of the experimental group, A_c_ (Control): Absorbance values of the control group, A_b_ (Blank): Absorbance values of the blank group.

### Elastase inhibition experiment

Elastase inhibitory activity was assessed spectrophotometrically. Reaction mixtures in 96-well plates contained 50 μL sample, 50 μL porcine elastase, and 100 μL substrate solution. After mixing and 5-min incubation at room temperature, absorbance was measured at 420 nm [22, 24]. Controls substituted samples with distilled water, while blanks replaced substrate with distilled water. All assays were conducted in triplicate, with inhibition rates calculated using formula (2).

2 $$Inhibition\;rate\left( \% \right) = 1 - \left( {A_{n} - A_{n}^{\prime } } \right)/\left( {A_{0} - A_{0}^{\prime } } \right) \times 100\%$$

In the formula, A_n_: the absorbance of the sample solution, and A_n_′: the absorbance of the sample reference substance; A_0_: Absorbance of the blank well, A_0_′: Absorbance of the blank control well.

### Collagenase inhibition experiment

Collagenase inhibitory activity was assessed by measuring absorbance at 335 nm following substrate cleavage [24]. The assay mixture in a 96-well plate contained 50 μL sample, 50 μL Type I collagenase, 60 μL Tris–HCl buffer, and 50 μL water. After mixing and incubation at 37 °C for 30 min, 50 μL substrate solution was added, followed by additional 30-min incubation at 37 °C. Control wells were prepared by replacing sample with distilled water, and blank wells by replacing substrate with distilled water. All assays were conducted in triplicate, with inhibition rates calculated using formula (2).

### Type I collagen secretion-promoting experiment

Following 24 h incubation of HDFs seeded in a 24-well plate, the supernatant was discarded. The experimental groups were then exposed to 500 μL of the drug solution at different concentrations, formulated in basal medium, while a blank control group was established with five replicates for each. Post-treatment, the supernatant was collected after a further 24 h. Measurements of optical density at 450 nm were carried out in compliance with the instructions of a commercial human PINP ELISA kit. Collagen secretion levels were subsequently determined through the kit's standard curve.

### Preparation of composite hydrogel

Synthesis of single-component hydrogels: A DDAM-1 solution (50 mg/mL) was prepared as the matrix solution by magnetic stirring until complete dissolution. Subsequently, solutions of NMN (92 mg), DOMOS (1 g), and HA (1 g), each dissolved separately in 20 mL of pure water, were added to this matrix solution. The mixture was then stirred continuously at 80 °C for 30 min. Finally, the reaction mixture was allowed to stand at ambient temperature (25 °C) for 2 h to complete gelation, yielding the corresponding hydrogel.

Synthesis of the composite hydrogel: The DDAM-1/NMN matrix solution was prepared using the method described above. Under continuous stirring, DOMOS (1 g) and HA (1 g) were sequentially added to this solution. After the addition of all components, the mixture was stirred continuously at 80 °C until a homogeneous phase formed [25–27]. The system was then transferred to room temperature and left undisturbed for 2 h, resulting in the formation of the DDAM-1/NMN/DOMOS/HA composite hydrogel.

### Cryo-Scanning electron microscopy

Observe the microstructure of the composite hydrogel in solution state using Cryo-scanning electron microscopy (Cryo-SEM, Sigma 300, Zeiss, Germany). After pre-freezing the prepared sample in liquid nitrogen, sublimate at − 70 °C for 15 min, then section and analyze morphological characteristics.

### Rheological testing of hydrogel materials

Rheological characterization was conducted using a modular compact rheometer (MCR302, Anton Paar, Graz, Austria) with RheoCompass software for data acquisition and analysis, applied a frequency sweep from 0.1 to 10 Hz at a constant 5% shear strain and a temperature of 25 °C, utilizing a 25 mm diameter plate. The values for storage modulus (G′), loss modulus (G″), shear stress, and viscosity were subsequently obtained from the software.

### Isothermal titration calorimetry (ITC) experiments

Using an isothermal titration calorimeter (MicroCal PEAQ-ITC, Malvern, UK), 40 μL of NMN (1 mg/mL) from the injection syringe was titrated into the sample cell containing 260 μL of DOMOS/DDAM-1/HA (5 mg/mL), respectively. The titration was performed over 17 injections, with deionized water used as a reference for blank subtraction [28]. Data were analyzed and fitted using the MicroCal PEAQ-ITC Analysis software.

### Molecular docking

The three-dimensional structures of hyaluronic acid and nicotinamide (NMN) were retrieved from the PubChem database. Their initial geometries were optimized by energy minimization using the MM2 force field in Chem3D. Using the AutoDock Tools software package [29], hydrogen atoms were added, and Gasteiger charges were calculated for all molecules. NMN was defined as the ligand with its rotatable bonds set to default parameters, while the polysaccharide molecules (HA, DOMOS, DDAM-1) were treated as rigid receptors. Semi-flexible molecular docking was performed using the AutoDock Vina program [30]. The grid box for docking was centered on the geometric center of each polysaccharide molecule. The binding free energy (ΔG) for each resulting complex was calculated.

### Analysis of drug combination

HDFs were inoculated in 96-well plates and cultured for 24 h. The supernatant was discarded and diluted compounds DOMOS, DDAM-1, HA, NMN and Mix were added to treat the cells. At the same time, a blank control group was established as a reference. Five duplicate wells were set for each treatment group and cultured at 37 °C. The culture medium was removed at 24 h, MTS was detected and the absorbance value was measured according to the operating procedures, and the combination index was calculated according to the survival rate at different concentrations.

### Establishment of a D-galactose-induced human dermal fibroblast senescence model

After seeding in 96-well plates and adhering, cells were treated with various concentrations of D-galactose (10, 20, 40, 60, and 80 g/L) [31]. Cell viability was then evaluated by MTS assay at 24/48-h post-treatment, according to the manufacturer’s protocol.

### β-galactosidase staining

The extent of cellular senescence in HDFs was determined by SA-β-galactosidase staining [32]. After being seeded into 6-well plates, the cell cultures were subdivided into four separate test groups: control (normal medium), model (D-galactose treatment), L-Mix (100 μg/mL composite hydrogel), and H-Mix (400 μg/mL composite hydrogel). Following 24 h D-galactose induction (except control), the treatment groups were exposed to respective concentrations of the composite hydrogel (Mix) for 24 h. the cells were subjected to fixed and then incubated with the staining solution from a commercial SA-β-gal kit at 37 °C, adhering strictly to the procedure supplied by the manufacturer. Senescent cells were identified by the development of blue staining and observed under a light microscope.

### Reactive oxygen species (ROS) detection

Following culture and treatments outlined in Sect. 2.5, HDFs were washed with PBS and incubated with 10 μM DCFH-DA for 30 min to assess intracellular ROS levels [33]. Following two additional PBS washes, fluorescence was visualized using an inverted fluorescence microscope (excitation: 488 nm, emission: 525 nm). The fluorescence intensity was quantified with ImageJ software.

### SASP assay

Levels of IL-6 and MMP-3 in the culture supernatant were measured using enzyme-linked immunosorbent assay (ELISA). After a 24-h treatment in 24-well plates (Sect. 2.5), supernatants were harvested and analyzed in accordance with the manufacturer’s instructions, using a microplate reader to detect absorbance at 450 nm.

### Animal experimental design

#### D-galactose induces aging in mice

All animal experiments were conducted in compliance with the guidelines approved by the Animal Ethics Committee of Kunming Institute of Botany, Chinese Academy of Sciences (Approval No: kib202410001). Eight-week-old male C57BL/6 J mice (20 ± 2 g) were acclimatized for one week and randomly allocated into four groups (n = 10): normal control, model, low-dose composite hydrogels (L-Mix, 50 mg/kg/day), and high-dose composite hydrogels (H-Mix, 100 mg/kg/day). The model, L-Mix, and H-Mix groups received daily intraperitoneal injections of D-galactose (200 mg/kg) for 8 weeks to induce aging [34], while the control group was administered 0.9% saline. From the fourth week of modeling, hair removal was performed on the dorsal skin of the mice, and the L-Mix and H-Mix groups began topical application of compound hydrogel at corresponding doses on the dorsal area for treatment, the control group and the model group were administered sterile PBS applied topically to the skin surface. All mice were maintained under standard laboratory conditions with ad libitum access to food and water. After 8 weeks, following a 12-h fast, the mice were anesthetized, and dorsal skin samples were collected, rinsed with saline, and stored in liquid nitrogen for subsequent analysis. Successful aging modeling was confirmed by observed phenotypes (lethargy, reduced activity, coarsened fur, skin thinning and laxity, weight loss) [35] and histologically verified reductions in skin associated dermal thickness.

#### The level of MMP3 in skin tissue was detected by ELISA

Dorsal skin tissues were isolated and carefully dissected to remove adjacent non-cutaneous components. The supernatant was collected after centrifugation of the lysed samples for subsequent analysis. Total protein concentration was determined using a BCA protein assay kit according to the manufacturer’s protocol, with all samples adjusted to a uniform concentration using lysis buffer. MMP-3 levels were quantified using a commercial ELISA kit, with absorbance measurements performed in accordance with the manufacturer’s instructions.

#### H&E and Sirius red staining

Skin wounds and adjacent tissues were harvested, sectioned, and subjected to hematoxylin–eosin (H&E) and Sirius Red staining. The stained sections were then examined under a light microscope for imaging, and epidermal thickness as well as collagen area were quantified using ImageJ software.

#### Immunohistochemical analysis

Immunohistochemical staining was conducted on sections obtained from paraffin-embedded tissue samples. Visualization and image acquisition were performed using standard light microscopy.

#### Western blot analysis

Four separate test groups and one Positive group receiving HA + NMN at a concentration of 50 mg/mL used to Western blot analysis. Proteins were extracted from HDFs with RIPA lysis buffer. After concentration measurement by BCA assay, equal protein loads were separated and electro transferred onto PVDF membranes. Blocking was conducted with 5% skim milk-TBST for 1 h at room temperature, followed by an overnight incubation at 4 °C with primary antibodies against SIRT1 (1: 1000), TGF-β (1: 1000), SMAD3 (1: 1000), FOXO3 (1: 1000), and GAPDH (1: 5000). After exposure to suitable HRP-conjugated secondary antibodies, the membranes were developed with an enhanced chemiluminescence substrate. Band quantification was performed from three independent experiments using ImageJ.

### FOXO inhibitor treatment

In the FOXO3 inhibition experiment, after the cells were seeded in a 6-well plate, except for the blank control group, HDFs were first added with JY-2 and D-gal at the same time for 24 h. The concentration of JY-2 was 40 μM and the concentration of D-gal was 40 g/L. The concentration was determined based on preliminary experiments and previous research results. After the treatment was completed, the Mix group and the JY-2 + Mix group were treated with 400 μg/mL composite hydrogel for 24 h, and the HDFs cells in each group were collected and analyzed by Western blot according to the experimental method of 2.16.5. After the addition of inhibitors, type I collagen secretion, β-galactosidase analysis and SASP analysis were performed in Sects. 2.5, 2.13, 2.15.

### Statistical analyses

All data were analyzed with Microsoft Excel 2019 and GraphPad Prism 9 software (GraphPad, San Diego, CA, USA), and are expressed as t the mean ± SD (standard deviation) of triplicate determinations. We used the *t* test and one-way ANOVA for significance testing, *P* < 0.05 was considered statistically significant.

## Results and discussion

### Primary screening for anti-ageing activity

Based on established roles of nicotinamide and hyaluronic acid in anti-aging research [36–38], this study selected four compounds for preliminary screening: HA, and NMN as well-established commercial agents, along with DOMOS and DDAM-1, which were previously identified by our group for their significant anti-aging activity and carrier properties, respectively. The anti-aging potential was evaluated by assessing the inhibitory effects on collagenase and elastase, as well as the promotion of type I collagen secretion in HDFs.

**Fig. 1:**
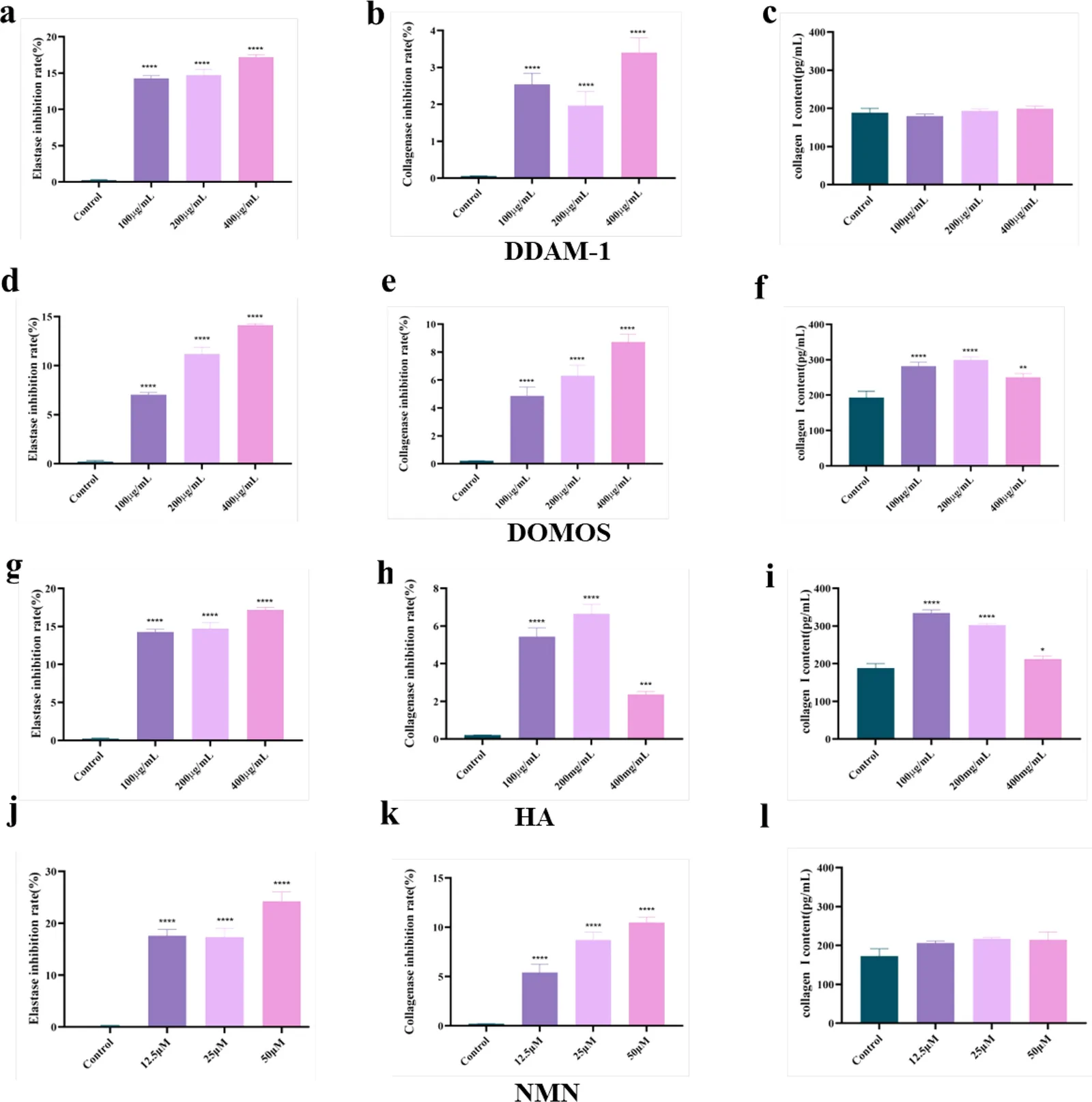
Anti-aging activity preliminary screening. **a**–**c** Anti-aging preliminary screening results of DDAM-1. **a** Inhibition results of DDAM-1 on elastase system. **b** Inhibition results of DDAM-1 on collagenase system. **c** The effect of DDAM-1 treatment on the expression level of type I collagen in HDFs. **d**–**f** Anti-aging preliminary screening results of DOMOS. **d** Inhibition results of DOMOS on elastase system. **e** Inhibition results of DOMOS on collagenase system. inhibition. **f** The effect of DOMOS treatment on the expression level of type I collagen in HDFs. **g**–**i** Anti-aging preliminary screening results of HA. **g** Inhibition results of HA on elastase system. **h** Inhibition results of HA on collagenase system. inhibition. **i** The effect of HA treatment on the expression level of type I collagen in HDFs. **j**–**l** Anti-aging preliminary screening results of NMN. **J** Inhibition results of NMN on elastase system. **k** Inhibition results of NMN on collagenase system. inhibition. **l** The effect of NMN treatment on the expression level of type I collagen in HDFs. Data are presented as means ± SD. *n* = 3, compared to the control group *p < 0.05, **p < 0.01, ***p < 0.001 and ****p < 0.0001

The cytotoxicity of the four compounds was assessed using the MTS assay (Additional file 1: Fig. S2), which showed no significant cytotoxicity within the tested concentration range. Further analysis using spectrophotometry revealed that DDAM-1, DOMOS, HA, and NMN all significantly inhibited the activities of elastase and collagenase (Fig. 1a–l). Among them, NMN and DOMOS exhibited dose-dependent inhibitory effects on both enzymes. In addition, type I collagen secretion in HDFs was measured by ELISA.

The results indicated that treatment with DOMOS and HA significantly promoted type I collagen expression compared to the control group. In contrast, DDAM-1 did not enhance collagen secretion, and no significant change was observed in the NMN-treated group. The latter finding may be associated to the potential skin irritation caused by NMN under certain conditions. In summary, the preliminary activity screening demonstrated that NMN primarily exerts its anti-aging effects by inhibiting elastase and collagenase, whereas the polysaccharides DOMOS and HA function mainly by promoting the secretion of type I collagen (Fig. 1f, i).

### Preparation and characterization of composite hydrogels

Based on the carrier properties of DDAM-1, hydrogels were prepared by combining it with DOMOS, HA, and NMN, respectively. Results indicated that only the combination of DDAM-1 and NMN successfully formed a hydrogel (Additional file 1: Fig. S3). Therefore, DOMOS and HA were further incorporated into the DDAM-1/NMN hydrogel system to construct a composite hydrogel (denoted as Mix), whose structure and properties were systematically characterized.

**Fig. 2:**
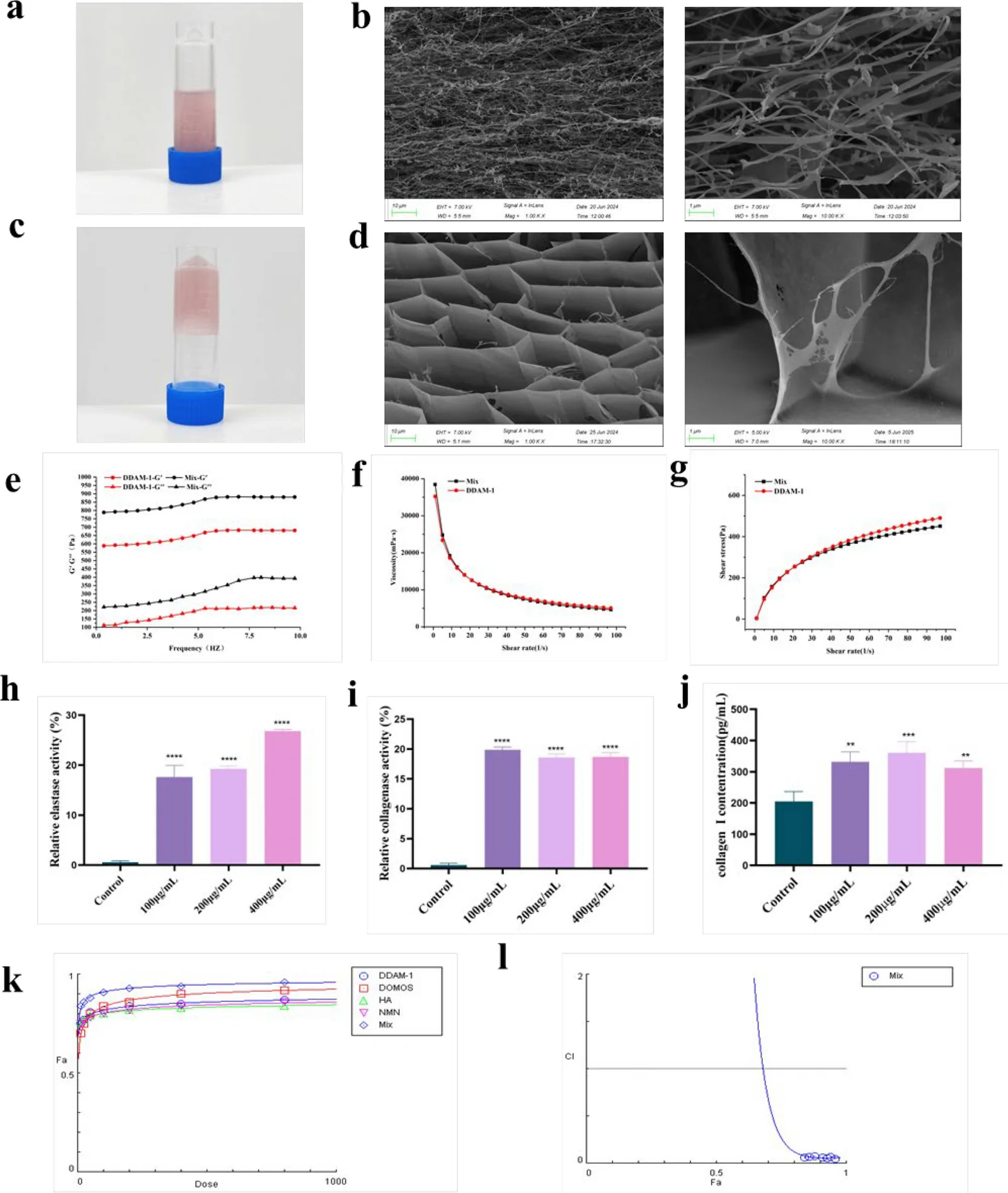
Preparation Characterization and Anti-aging Activity of Composite Hydrogel. **a** Image of DDAM-1 aqueous solution. **b** Cryo-SEM image of DDAM-1 at a scale of 10 μm (left) and 1 μm (right). **c** Image of composite hydrogel formation. **d** Cryo-SEM image of composite hydrogel at a scale of 10 μm (left) and 1 μm (right). **e** Energy storage modulus diagram of composite hydrogel. **f** Viscosity diagram of composite hydrogel. **g** Shear stress curve of composite hydrogel. **h** Results of the Elastase Inhibition Experiment with Composite Hydrogels. **i** Results of Collagenase Inhibition Experiment with Composite Hydrogel. **j** After HDFs were treated with the composite hydrogel, the expression level of type I collagen was detected. **k** Drug dose–response curve; **l** Combination index (CI) of the complex; Data are presented as means ± SD. *n* = 3, compared to the control group *p < 0.05, **p < 0.01, ***p < 0.001 and ****p < 0.0001

Morphological and structural analyses revealed that the composite hydrogel exhibited superior mechanical properties (Fig. 2c) compared to the DDAM-1 alone (Fig. 2a). Cryo-scanning electron microscopy (Cryo-SEM) imaging showed that the composite hydrogel displayed a regular three-dimensional porous network structure (Fig. 2d), which was distinctly different from the original morphology of DDAM-1 (Fig. 2b), indicating a significantly increased cross-linking density. This structural feature endowed the hydrogel with enhanced deformation tolerance, reflecting the orderly arrangement of the loaded molecules and strengthened intermolecular interactions. Rheological tests further elucidated the film-forming ability (Additional file 1: Fig. S4) and mechanical properties (Additional file 1: Tables S1–S3, Fig. 2e–g) of the composite hydrogel. Throughout the frequency sweep, the storage modulus (G′) of both DDAM-1 and the composite hydrogel consistently exceeded the loss modulus (G″), with a notably higher G′ observed for the composite system (Fig. 2e), confirming the formation of a stable elastic network structure. Furthermore, the apparent viscosity decreased with increasing shear rate, demonstrating typical shear-thinning behavior (Fig. 2f, g), which indicates favorable injectability of the hydrogel. Collectively, the rheological results demonstrate that the composite hydrogel formed a denser, more stable, and robust three-dimensional cross-linked network.

The anti-aging efficacy of the composite hydrogel was further evaluated based on its inhibition of elastase and collagenase activities and its promotion of type I collagen secretion. Cytotoxicity assays confirmed that the composite hydrogel exhibited no significant cytotoxicity within the tested concentration range (Additional file 1: Fig. S5). Anti-aging performance results showed that the composite hydrogel significantly enhanced the inhibition rates of both elastase and collagenase compared to the control group (Fig. 2h, i). Moreover, HDFs treated with the composite hydrogel exhibited a marked increase in type I collagen secretion across three tested concentrations (Fig. 2j). In summary, compared to individual components, the composite hydrogel demonstrated superior performance across multiple anti-aging indicators, indicating that component integration achieves a synergistic enhancement, and the composite system exhibits significantly greater anti-aging potential (Additional file 1: Table S6). In order to verify whether there is a synergistic effect in the preparation of composite hydrogels, the combined drug index analysis was performed on the HDFs cell survival rate data. The analysis results are shown in Fig. 2l and Additional file 1: Table S4. The CI value of the composite hydrogel at each concentration is between 0.04589 and 0.07811, far less than 0.5, indicating that the composite hydrogel prepared by the combination of four single components DDAM-1, DOMOS, HA, and NMN has a strong synergistic effect. The dose–response curve results are shown in Fig. 2k. Compared with the use of these drugs alone, the complex can produce greater effects at a lower total dose. As shown in Additional file 1: Table S5, the data of all drugs and mixed use showed a good linear relationship (r > 0.99), indicating that the dose–effect relationship was well fitted and the data were highly reliable.

### Gelation mechanism of the composite hydrogel

**Fig. 3:**
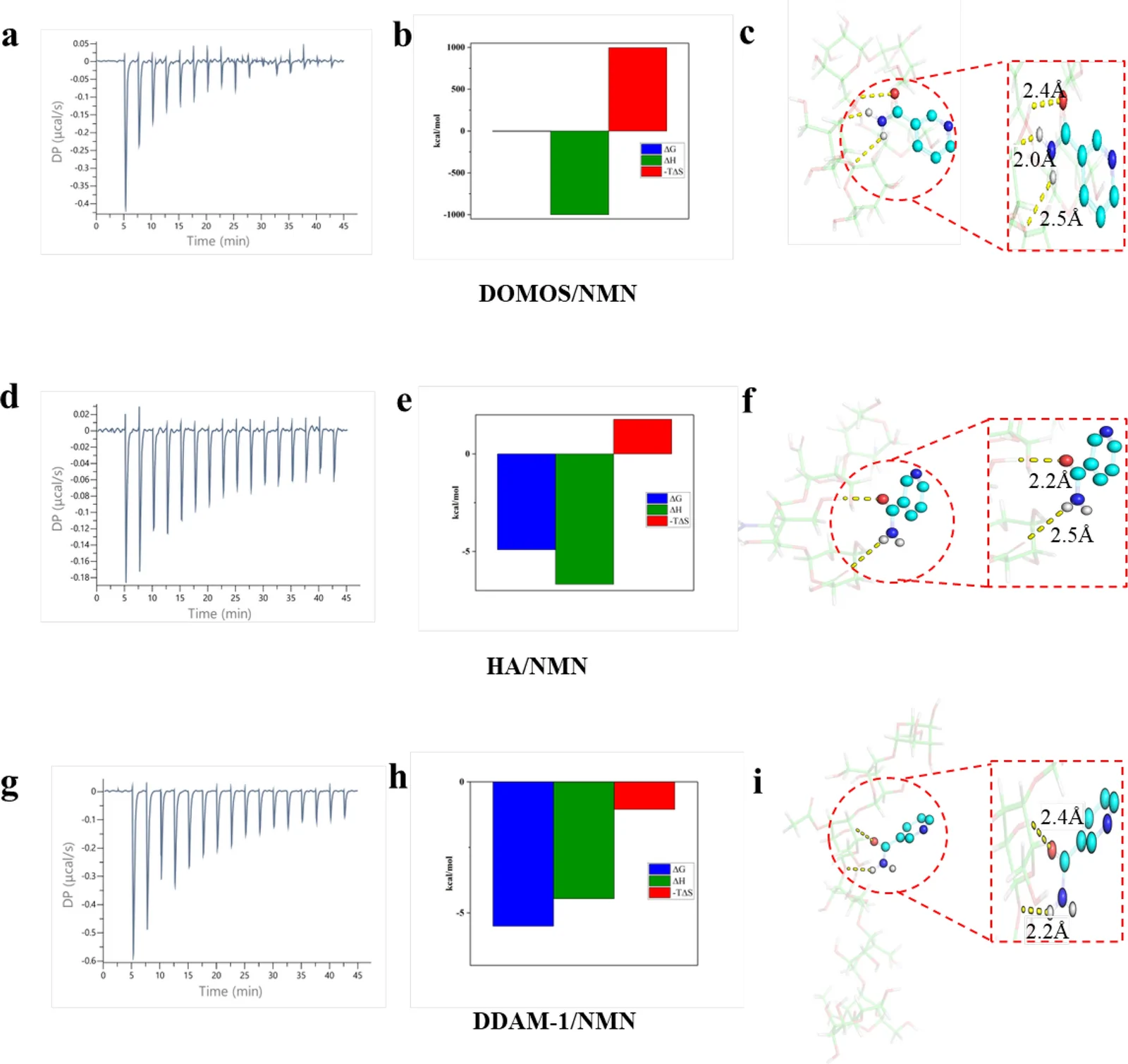
Mechanism diagram of composite hydrogel formation. **a** Standard calorimetric titrations of 5 mg/mL DOMOS into 1 mg/mL NMN solution at 25 °C. **b** The fitting data of ITC from DOMOS/NMN complexes; ΔG, ΔH, − TΔS represented Gibbs free energy of binding (blue), enthalpy changes (green), and entropy changes (red). **c** Molecular docking diagram of DOMOS and NMN. **d** Standard calorimetric titrations of 5 mg/mL HA into 1 mg/mL NMN solution at 25 ℃. **e** The signature of ITC from HA/NMN. **f** Molecular docking diagram of HA and NMN. **g** Standard calorimetric titrations of 5 mg/mL DDAM-1 into 1 mg/mL NMN solution at 25 °C. **h** The signature of ITC from DDAM-1/NMN. **i** Molecular docking diagram of DDAM-1 and NMN

To elucidate the interaction mechanisms among the components within the composite hydrogel, isothermal titration calorimetry (ITC) and molecular docking simulations were employed. ITC experiments provided key thermodynamic parameters of the binding process, including the dissociation constant (KD), binding stoichiometry (N), Gibbs free energy change (ΔG), entropy change (− TΔS), and enthalpy change (ΔH) (Additional file 1: Figs. S6–S8, Table S7). The results demonstrated that DOMOS (Fig. 3a, b), HA (Fig. 3d, e), and DDAM-1 (Fig. 3g, h) all bind to NMN, a negative ΔG value indicated that the binding between the molecules was spontaneous. The observation that ΔH < 0 and − TΔS > 0 suggested that the binding of both DOMOS and HA to NMN exhibited a typical enthalpy-driven profile. Their co-assembly process was therefore primarily mediated by hydrogen bonding and/or van der Waals interactions, rather than by entropy-dominated hydrophobic effects. In contrast, the combination of ΔH < 0 and − TΔS < 0 for the binding of DDAM-1 to NMN revealed an enthalpy–entropy co-driven mechanism, with enthalpy serving as the dominant contributor. These thermodynamic results reflect the formation of specific and stable associative structures between the molecules.

Furthermore, molecular docking simulations revealed the specific binding modes (Fig. 3, Additional file 1: Table S8). In the DOMOS-NMN interaction (Fig. 3c), extensive van der Waals contacts and polar interactions were observed between the helical region of the polysaccharide chain and the NMN molecule. The HA-NMN complex (Fig. 3f) primarily formed hydrogen bonds between the carboxyl groups of HA and the amide group of NMN. In contrast, the binding of DDAM-1 to NMN (Fig. 3i) relied on a hydrogen-bonding network established between the hydroxyl groups of DDAM-1 and the nitrogen-containing heterocycle of NMN.

In summary, both ITC and molecular docking results consistently confirmed a strong binding affinity between the two Dendrobium polysaccharides (DDAM-1 and DOMOS) and NMN, with hydrogen bonding and van der Waals forces serving as the dominant interactions. This conclusion provides crucial molecular-level theoretical support for the construction of polysaccharide-NMN-based composite hydrogel systems.

### *The *in vitro* anti-aging effects of composite hydrogels*

**Fig. 4:**
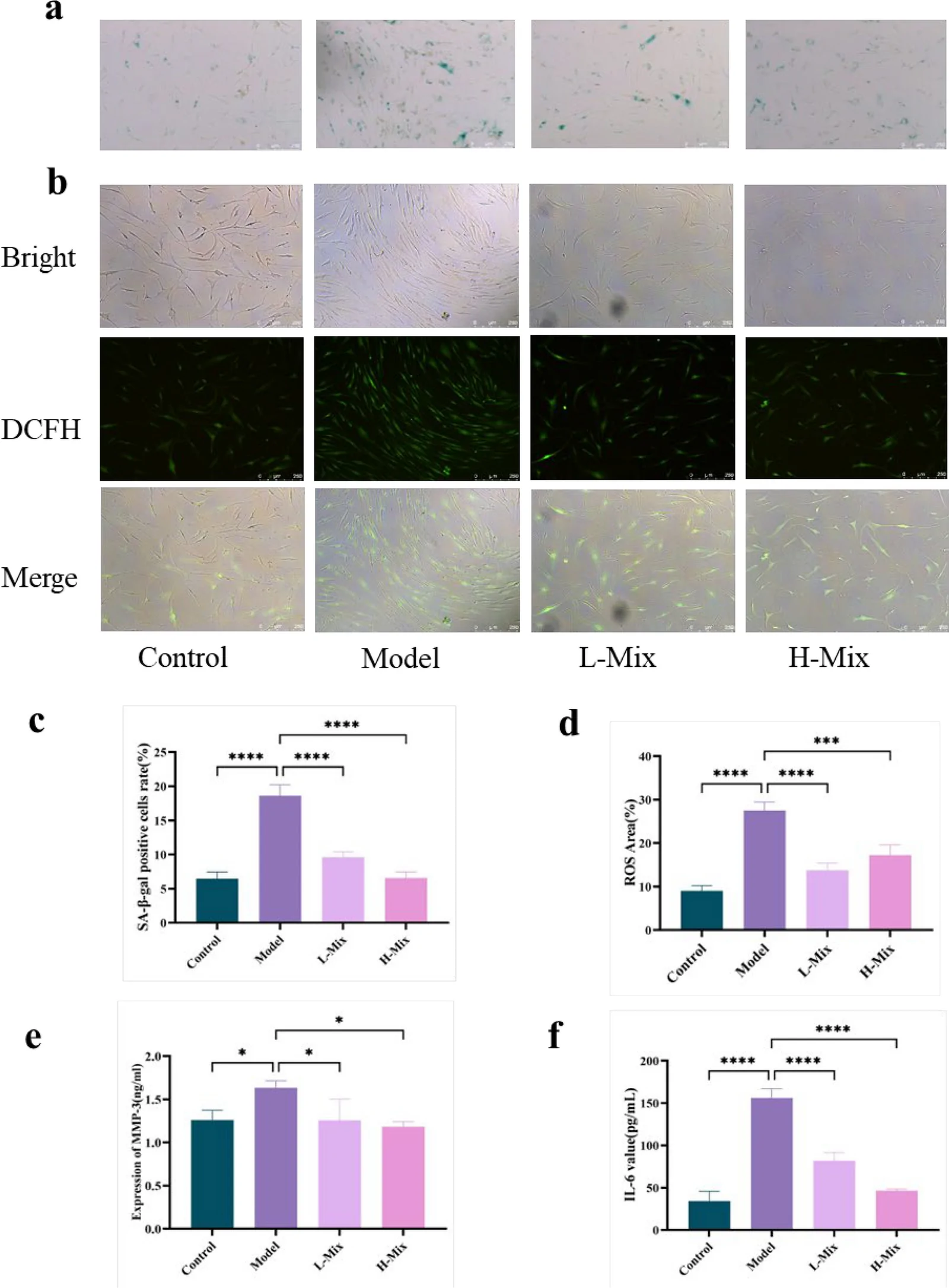
Anti-aging effects of composite hydrogels in vitro. **a** β-galactosidase levels in HDFs, induced by 40 g/L D-galactose and treated with varying concentrations of the composite hydrogel for 24 h, were visualized by staining. **b** Intracellular ROS levels were measured by DCFH-DA staining under the same treatment conditions. **c**, **d** Quantitative analysis of the percentage of SA-β-gal and ROS-positive HDFs, respectively. **e**, **f** The expression levels of MMP-3 and IL-6 in HDFs under the aforementioned conditions were assessed using ELISA kits. Data are presented as means ± SD. *n* = 3, compared to the model group *p < 0.05, **p < 0.01, ***p < 0.001 and ****p < 0.0001

Previous studies have demonstrated that high concentrations of D-galactose can significantly elevate intracellular reactive oxygen species (ROS) levels [39, 40] strongly induce the expression and secretion of the senescence-associated secretory phenotype (SASP) [41], and ultimately trigger cellular senescence [42]. In this study, a senescence model was established in HDFs using D-galactose induction. Based on cytotoxicity assay results, a concentration of 40 g/L D-galactose was selected as the optimal concentration for modeling (Additional file 1: Fig. S9). Assessment of cellular senescence using β-galactosidase staining demonstrated a considerable increase in SA-β-gal-positive cell numbers in the model group versus the normal control (Fig. 4a, c), verifying the successful replication of the senescence model. Intervention with the composite hydrogel notably reduced the SA-β-gal positivity rate, suggesting its potential to delay HDFs senescence.

Furthermore, intracellular ROS levels were detected using a fluorescent ROS probe (Fig. 4b, d). Faint fluorescence was observed in the normal control group, indicating a physiological state of low ROS. In contrast, the model group exhibited a significant enhancement in ROS fluorescence intensity. Treatment with the composite hydrogel effectively reversed this trend, significantly reducing ROS levels. Excessive ROS also influences the secretion of SASP factors. ELISA results revealed that, compared to the model group, treatment with the composite hydrogel significantly downregulated the expression levels of MMP-3 and IL-6 in HDFs (Fig. 4e, f). In summary, the composite hydrogel effectively mitigates D-galactose-induced HDFs senescence by inhibiting excessive ROS accumulation and modulating the expression of key SASP factors.

### The in vivo anti-aging effects of composite hydrogels

**Fig. 5:**
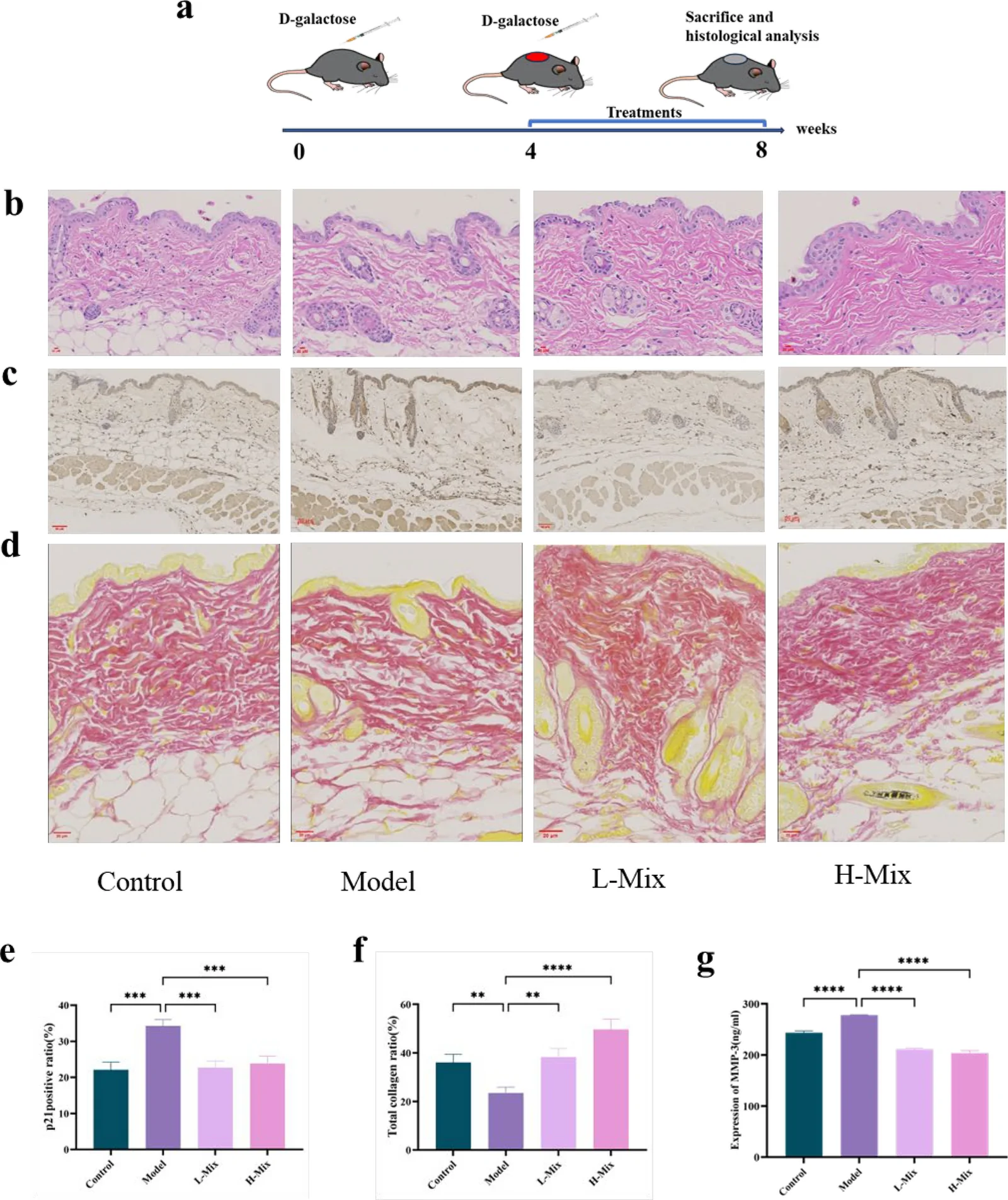
The anti-aging effects of composite hydrogel in vivo. **a** Diagram of animal model development and intervention. **b** Evaluation of the histopathological alterations in mouse skin across different groups via Hematoxylin and Eosin (H&E) staining. Scale bar = 20 μm. **c** Assessment of skin aging changes in mice across different groups using P21 immunohistochemical staining. Scale bar = 50 μm. **d** Assessment of overall collagen expressed in skin tissues of different mouse groups through Sirius Red staining analysis. Scale bar = 50 μm. **e** Quantitative analysis of p21 protein expression in skin tissue sections from mice in each group was performed using ImageJ software. **f** Quantitative analysis of collagen expression in skin tissue sections from mice in each group was performed using ImageJ software. **g** Quantification of MMP-3 levels in mouse skin homogenates using the ELISA specific kit. Data are presented as means ± SD, compared to the model group *p < 0.05, **p < 0.01, ***p < 0.001 and ****p < 0.0001

To evaluate the protective effect of the composite hydrogel on skin structure, histopathological analysis was performed on dorsal skin tissues of mice. Hematoxylin and eosin (H&E) staining results (Fig. 5b) revealed that, compared with the normal group, the D-galactose-induced aging model group exhibited an irregular and unevenly thickened epidermis, a thinner dermis, inflammatory cell infiltration, and disorganized collagen fiber structure. Treatment with the composite hydrogel (Mix) significantly reversed these aging-related pathological changes, resulting in a more regular epidermal morphology and a notable increase in dermal thickness.

The p21 protein has been established as a biomarker of cellular senescence, and its clearance has been shown to alleviate aging [43]. In this study, immunohistochemical staining was used to detect p21 expression levels in skin tissues (Fig. 5c, e). The results demonstrated a significant upregulation of p21 expression in the skin of the model group, while intervention with the composite hydrogel effectively reduced p21 expression, suggesting its potential to delay the skin aging process.

During skin aging, the content and structure of collagen fibers in the dermis undergo significant degradation. As shown by Sirius Red staining results (Fig. 5d, f), the skin tissue of the normal control group displayed an intact structure with dense and uniformly arranged collagen fibers. In contrast, the model group showed markedly reduced collagen expression with loose and disordered fiber arrangement. After treatment with the composite hydrogel, both the density and organization of collagen fibers were significantly improved, indicating that the hydrogel effectively promotes collagen synthesis and orderly deposition.

Matrix metalloproteinases (MMPs) represent a family of proteolytic enzymes that are primarily responsible for catalyzing the breakdown of extracellular matrix (ECM) components, play a crucial role in skin aging [44, 45]. Among them, MMP-3 not only degrades type I and III collagen but also extensively disrupts other matrix components and activates other MMPs [35]. ELISA was used to measure MMP-3 expression levels in mouse skin tissues (Fig. 5g). The results showed that MMP-3 expression was significantly elevated in the model group, whereas treatment with the composite hydrogel markedly suppressed its expression. These findings suggest that the composite hydrogel may exert its anti-skin aging effects by downregulating MMP expression, reducing collagen degradation, and thereby synergistically promoting collagen homeostasis.

### Mechanism of anti-aging effects of the composite hydrogel

**Fig. 6:**
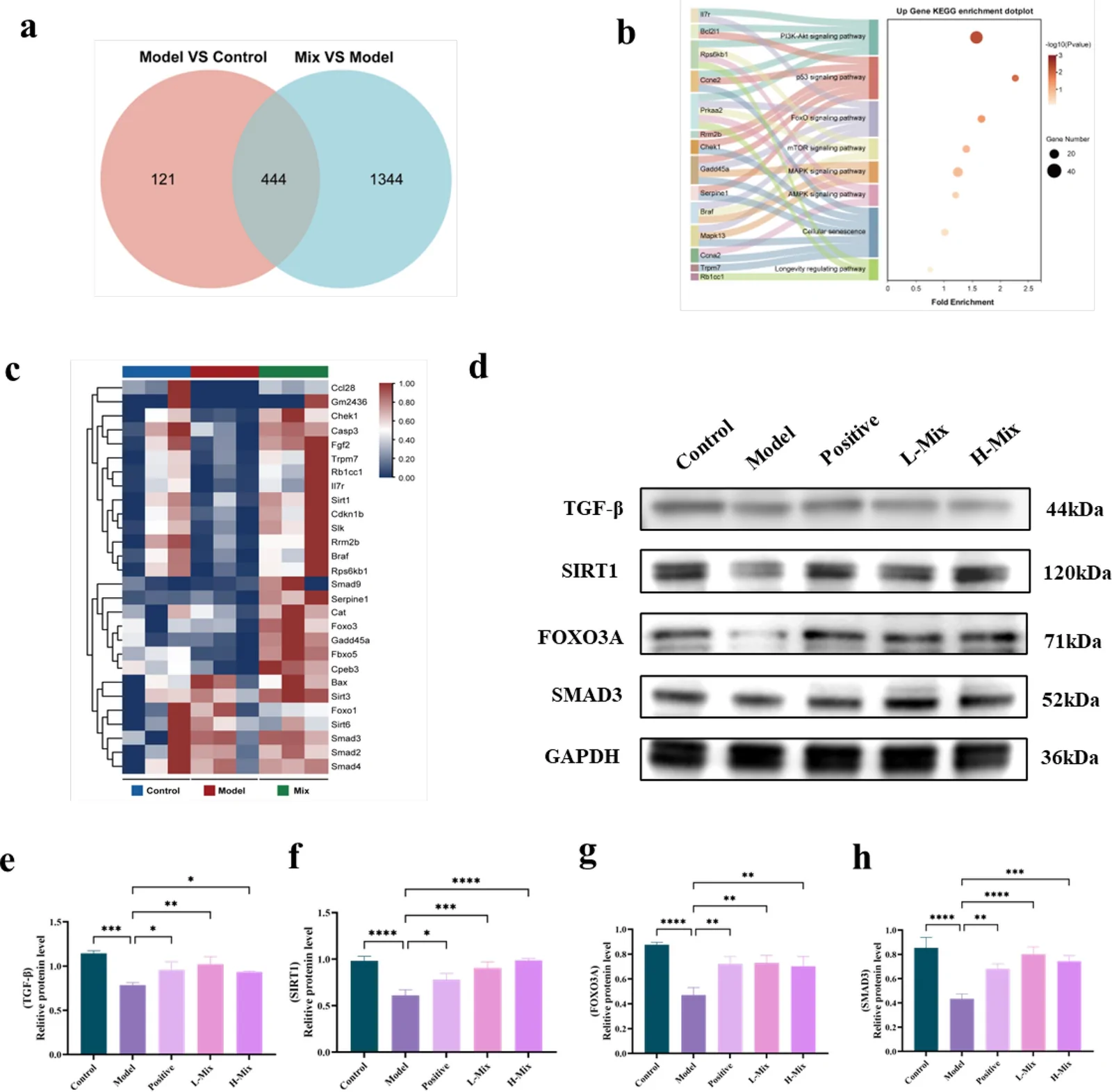
Anti-aging Mechanism of Composite Hydrogels. **a** Venn diagram of differentially expressed genes among groups. **b** Enrichment analysis plot of pathways with significant differences between groups. **c** KEGG enrichment analysis of differentially expressed genes. **d** Western blot analysis of the expression levels of SIRT1, SMAD3, TGFβ, and FOXO3. **e**–**h** Quantification of Western blots shown in **d** by ImageJ. Data are presented as means ± SD. *n* = 3, compared to the model group *p < 0.05, **p < 0.01, ***p < 0.001 and ****p < 0.0001

To further elucidate the upstream signaling pathways mediating the anti-aging effects of the composite hydrogel, high-throughput RNA sequencing was performed to compare the transcriptomic profiles of human dermal fibroblasts (HDFs) before and after model induction and treatment According to KEGG enrichment results, gene sets related to the FOXO signaling pathway were notably altered by treatment with the composite hydrogel (Fig. 6a, b, Additional file 1: Figs. S10–S13), which is well-established to play a crucial role in regulating cell survival, apoptosis, and longevity [46, 47]. To investigate whether the FOXO signaling pathway contributes to the anti-aging activity of the composite hydrogel, to investigate pathway-related protein expression, immunoblot analysis was performed. We focused on measuring the abundance of TGF-β, Smad3, SIRT1 and FOXO3, as TGF-β and Smad family members play a fundamental role in the regulation of cell growth and differentiation. The results demonstrated that treatment with the composite hydrogel significantly upregulated the expression of TGF-β, Smad3, SIRT1, and FOXO3 proteins compared to the control group (Fig. 6d–h). These findings indicate that the anti-aging effect of the composite hydrogel is mediated through the activation of the FOXO signaling pathway, thereby enhancing the expression of extracellular matrix (ECM) components.

### The composite hydrogel inhibits skin aging through the FOXO pathway

**Fig. 7:**
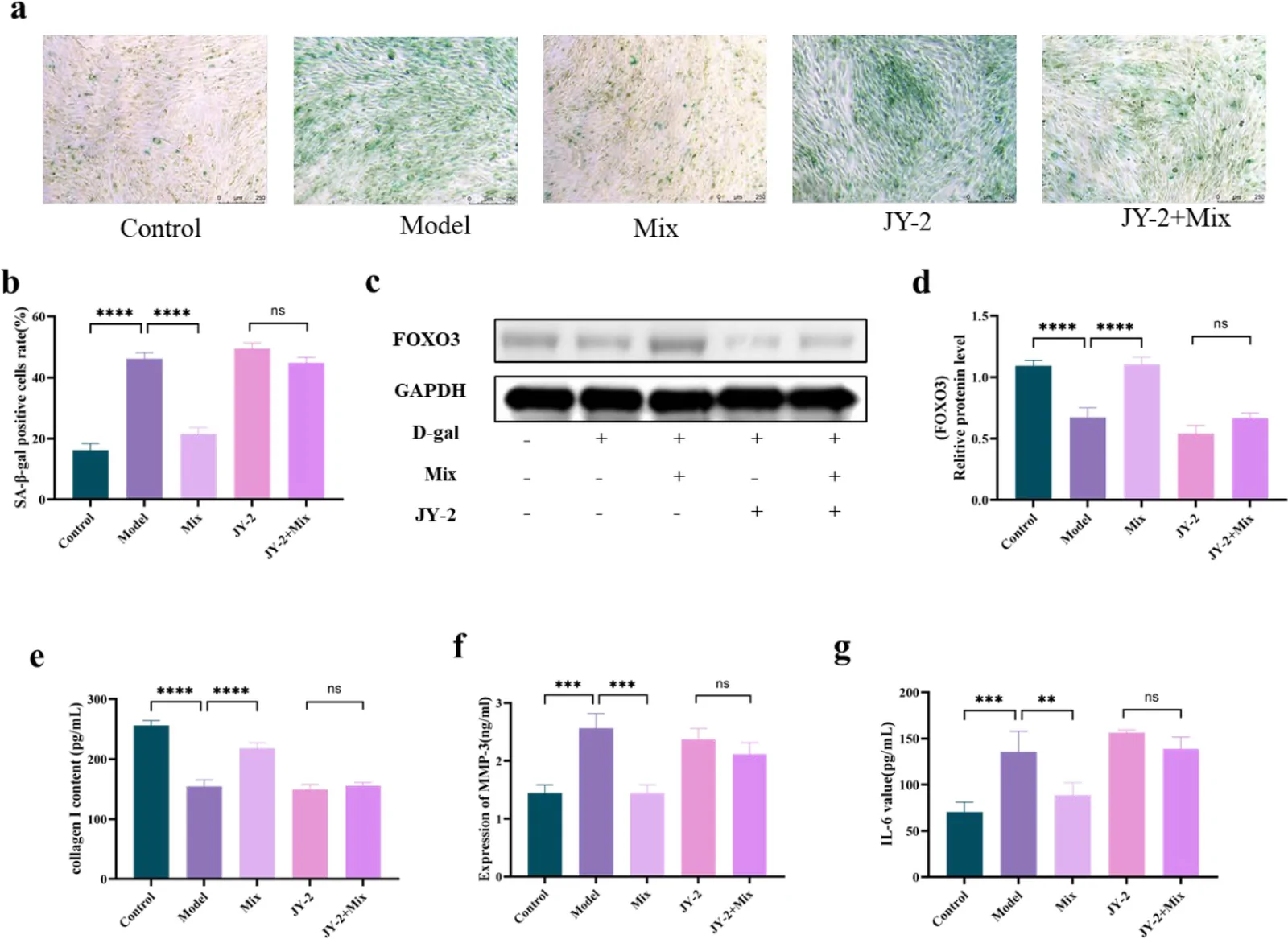
Composite hydrogel protects HSF cells from D-galactose-induced senescence through the FOXO signaling pathway. **a** The effect of composite hydrogel on β-galactosidase was observed after pretreatment of senescent HDFs cells with 40 μM JY-2. **b** Quantitative analysis of the percentage of SA-β-gal positive HDFs. **c** After pretreatment of senescent HDFs cells with 40 μM JY-2, the effect of the composite hydrogel on the FOXO signaling pathway was verified by Western blot. **d** ImageJ quantitative analysis was performed on the Western blot results shown in **c**. **e** After pretreatment of senescent HDFs cells with 40 μM JY-2, the effect of the composite hydrogel on the expression level of type I collagen was detected. **f** The effect of the composite hydrogel on the expression level of MMP-3 was detected after the senescent HDFs cells were pretreated with 40 μM JY-2. **g** The effect of composite hydrogel on the expression level of IL-6 was detected after pretreatment of senescent HDFs cells with 40 μM JY-2. Data are presented as means ± SD. *n* = 3, compared to the model group *p < 0.05, **p < 0.01, ***p < 0.001 and ****p < 0.0001

In order to further clarify the role of FOXO3 in the anti-skin aging of the composite hydrogel, HSF cells were pretreated with FOXO3 inhibitor JY-2 for verification [48], and various aging-related indicators were detected to evaluate whether the composite hydrogel could improve the aging of HSF cells through FOXO3. The results of β-galactosidase staining (Fig. 7a, b) are shown in the figure. There were fewer positive (blue) cells in the control group. Compared with the control group, the positive cells in the model group increased significantly, indicating that the β-galactosidase activity increased. Compared with the control group, the JY-2 group showed the same number of SA-β-gal positive cells as the model group, and the JY-2 + Mix group did not significantly improve the aging caused by D-galactose. In order to further verify the role of FOXO3 in the promotion of collagen synthesis by composite hydrogels, the effect of FOXO3 on the secretion of type I collagen was detected (Fig. 7e). ELISA results showed that the secretion of type I collagen in HSF cells after D-gal induction was significantly lower than that in the normal group. To 154.39 pg/mL, after 24 h of treatment with composite hydrogels, the level of collagen secretion increased significantly to 217.68 pg/mL. However, JY-2 treatment further aggravated the collagen loss in the model group, and the pro-collagen secretion effect of the composite hydrogel was significantly weakened. The collagen level in the JY-2 + Mix group was reduced to a level similar to that in the model group. In addition, the expression trends of aging-related secretory phenotypes IL-6 and MMP-3 were comparable to those of the model group (Fig. 7f, g). These data indicate that both D-galactose and JY-2 inhibit FOXO3 and accelerate aging, while the composite hydrogel alleviates D-galactose-induced aging at least in part by regulating the FOXO pathway. The effect on FOXO3 protein was shown in Fig. 7c. Compared with the control group, the JY-2 intervention group showed the same level of FOXO3 protein expression as the model group, while the addition of JY-2 intervention and the composite hydrogel could not up-regulate the level of FOXO3 protein, which also showed the same level of FOXO3 protein as the model group. In summary, the composite hydrogel alleviates D-galactose-induced HSF cell senescence at least in part by activating the FOXO signaling pathway.

## Conclusion

Above all, to cope with the complexity of skin aging, this study aims to construct a composite hydrogel via synergistic interactions to fortify the anti-aging activity. A novel composite hydrogel was successfully prepared successfully by Dendrobium polysaccharides (DDAM-1 and DOMOS), HA, and NMN. Originally, a polysaccharide-based hydrogel with a stable 3D porous network structure was prepared by self-assembly via hydrogen bonding and van der Waals force of DDAM-1, DOMOS, and NMN. In addition, ITC and molecular docking confirmed that NMN could further linked to HA which has anti-aging activity, forming a co-assembled composite hydrogel through hydrogen and oxygen atoms on amide bonds. The comprehensive in vitro and in vivo experiments be aimed to elucidate the repel skin aging activity and mechanisms of the composite hydrogel. In vitro assessments demonstrated that the composite hydrogel markedly inhibited the activities of elastase and collagenase and substantially promoted the secretion of type I collagen in human dermal fibroblasts (HDFs). In a D-galactose-induced cellular senescence model, the composite hydrogel exhibited potent cytoprotective effects by effectively scavenging excess ROS, reducing SA-β-gal activity, and downregulating the expression of key SASP factors, including IL-6 and MMP-3. The treatment with the composite hydrogel effectively mitigated aging-associated histopathological alterations, such as increased the thickness of epidermal and dermal, enhanced the density of collagen fiber in skin. The mechanism of anti-aging was via the activation of the FOXO pathway, as indicated by the upregulation of FOXO3, TGF-β, Smad3, and SIRT1 protein expressions. The successful construction of co-assembled composite hydrogels provides a novel coping strategy for the complexity of skin aging. Delaying skin aging is of great significance for maintaining people’s physical and mental health, ranging from aesthetics to healthy development.

## Supplementary Information

Additional file 1.

## Data Availability

Data will be made available on request.
