# Supplementary material for: A self-assembled Dendrobium polysaccharide hydrogel with anti-aging activity via the FOXO signaling pathway
Source: Nat Prod Bioprospect. 2026 Oct 1;16(1):89. doi: 10.1007/s13659-026-00642-y (PMC13627647; doi:10.1007/s13659-026-00642-y)
Supplement: Supplementary file 1 [file 13659_2026_642_MOESM1_ESM.docx]

Supplementary data for

**A Self-Assembled Dendrobium Polysaccharide** **Hydrogel** **with Anti-aging activity via the FOXO signaling pathway**

Yanye Zhang ^a, b,1^, Yuanhe Wang ^b,1^, Huiyan Shao ^b^, Xingli Guo ^b^, Pengcheng Xu ^b^, Yanlang Li ^b^, Yi Zeng ^b^, Yina Yang ^b^, Jiatao Guo ^b^, Jianke Jia ^b^, Fawu Dong ^a, *^, Jiang-miao Hu ^b, *^, Liu Yang^b, *^.

a Yunnan Key Laboratory of Chinese Medicine Processing, College of Chinese Materia Medica, Yunnan University of Chinese Medicine, Kunming, 650500, China.

b Key Laboratory of Phytochemistry and Natural Medicines, and Key Laboratory of Phytochemistry and Plant Resources in West China, Kunming Institute of Botany, Chinese Academy of Sciences, Kunming, Yunnan, 650201, China.

*** Corresponding Author**

Tel.: +86-871-65223264; E-mail address: [yangliu@mail.kib.ac.cn](mailto:yangliu@mail.kib.ac.cn) (L. Yang); [hujiangmiao@mail.kib.ac.cn](mailto:hujiangmiao@mail.kib.ac.cn) (J.M. Hu); [yucmlxs@163.com](mailto:yucmlxs@163.com) (F.W. Dong).

^1^ Y. Y. Z. & Y.H. W. contributed equally to this work.

**Fig S1.** Structure of DDAM-1(a) and DOMOS(b).

**Fig.S2** The impact of compounds on cellular viability. (a) HA. (b)DDAM-1. (c)DOMOS. (d) NMN.

**Fig.S3** Preparation process of hydrogel.

**Fig.S4** Molding properties of composite hydrogels.

**Table S1.** Composite hydrogel energy storage modulus graph data table.

| Frequency (HZ) | | G′G′′(Pa) | | | |
| --- | --- | --- | --- | --- | --- |
|  | DDAM-1-G′ | | Mix-G′ | DDAM-1-G′′ | Mix-G′′ |
| 0.375 | 588.312 | | 788.3127 | 112.713 | 221.7134 |
| 0.925 | 592.125 | | 792.1256 | 114.8176 | 224.8173 |
| 1.475 | 594.467 | | 794.4677 | 129.721 | 227.7213 |
| 2.025 | 598.323 | | 798.3239 | 134.3124 | 236.3124 |
| 2.575 | 605.514 | | 805.5142 | 143.1317 | 243.1315 |
| 3.125 | 611.678 | | 811.6787 | 155.6442 | 254.6442 |
| 3.675 | 621.421 | | 821.4215 | 168.2422 | 263.2422 |
| 4.225 | 634.637 | | 834.6376 | 182.332 | 284.3326 |
| 4.775 | 647.551 | | 847.5513 | 195.6821 | 295.6821 |
| 5.325 | 667.912 | | 867.9124 | 213.5754 | 314.5754 |
| 5.875 | 678.017 | | 878.0177 | 211.5921 | 334.5921 |
| 6.425 | 681.124 | | 881.1246 | 213.7334 | 353.7334 |
| 6.975 | 681.977 | | 881.9775 | 210.32 | 380.3222 |
| 7.725 | 681.235 | | 881.2356 | 217.23 | 397.234 |
| 8.075 | 680.124 | | 880.1244 | 218.3943 | 398.3943 |
| 8.625 | 680.135 | | 880.1354 | 218.6856 | 394.6856 |
| 9.175 | 680.196 | | 880.1963 | 216.1436 | 393.1436 |
| 9.725 | 680.333 | | 880.3338 | 216.4156 | 393.4156 |

**Table S2.** Viscosity data table of composite hydrogels.

| Shear rate(1/s) | Viscossity (mPa^.^s) | |
| --- | --- | --- |
|  | Mix | DDAM-1 |
| 1 | 3.8292 | 3.509 |
| 5 | 102.6 | 96.792 |
| 9 | 157.1 | 151.99 |
| 13 | 196.84 | 193.7 |
| 17 | 228.59 | 227.17 |
| 21 | 254.43 | 255.34 |
| 25 | 276.45 | 279.41 |
| 29 | 295.53 | 300.76 |
| 33 | 312.39 | 319.73 |
| 37 | 327.52 | 336.67 |
| 41 | 340.93 | 352.32 |
| 45 | 352.97 | 366.74 |
| 49 | 363.88 | 380 |
| 53 | 373.73 | 392.35 |
| 57 | 382.73 | 404.12 |
| 61 | 391.36 | 414.91 |
| 65 | 399.32 | 424.88 |
| 69 | 406.82 | 434.78 |
| 73 | 413.94 | 443.72 |
| 77 | 420.88 | 452.39 |
| 81 | 427.27 | 460.84 |
| 85 | 433.42 | 468.74 |
| 89 | 439.34 | 476.28 |
| 93 | 445.02 | 483.48 |
| 97 | 450.55 | 490.3 |

**Table S3.** Shear stress data table for composite hydrogels.

| Shear rate(1/s) | Shear stress (Pa) | |
| --- | --- | --- |
|  | Mix | DDAM-1 |
| 1 | 38453 | 35241 |
| 5 | 24810 | 23406 |
| 9 | 19225 | 18601 |
| 13 | 16125 | 15868 |
| 17 | 14072 | 13985 |
| 21 | 12547 | 12592 |
| 25 | 11370 | 11491 |
| 29 | 10424 | 10608 |
| 33 | 9645.3 | 9872.2 |
| 37 | 8992.2 | 9243.4 |
| 41 | 8426.6 | 8708 |
| 45 | 7932.7 | 8242.4 |
| 49 | 7498.1 | 7830.1 |
| 53 | 7109.7 | 7463.8 |
| 57 | 6761.9 | 7139.7 |
| 61 | 6454 | 6842.6 |
| 65 | 6174.5 | 6569.9 |
| 69 | 5921.1 | 6327.9 |
| 73 | 5690.4 | 6099.8 |
| 77 | 5481.8 | 5892.2 |
| 81 | 5287.2 | 5702.6 |
| 85 | 5108.2 | 5524.6 |
| 89 | 4943 | 5358.7 |
| 93 | 4789.7 | 5203.6 |
| 97 | 4647.4 | 5057.4 |

**Table S4.** Composite Index (CI) of Compounds.

| Total dose(μg/mL) | Fa | CI |
| --- | --- | --- |
| 12.5 | 0.84 | 0.06286 |
| 25 | 0.86 | 0.07040 |
| 50 | 0.88 | 0.07811 |
| 100 | 0.91 | 0.05972 |
| 200 | 0.93 | 0.05981 |
| 400 | 0.94 | 0.07140 |
| 800 | 0.90 | 0.04589 |

**Table S5.** Correlation Coefficients of Drugs

| Drug/Combo | r (correlation coefficient) |
| --- | --- |
| DDAM-1 | 0.99107 |
| DOMOS | 0.99765 |
| HA | 0.99619 |
| NMN | 0.99292 |
| Mix | 0.99159 |

**Table S6.** Comparison table of anti-aging activities of single compounds and of composite hydrogel.

| Sample name | Average elastase inhibition rate（%） | Average collagenase inhibition rate（%） | Increase rate of collagen secretion（%） |
| --- | --- | --- | --- |
| DOMOS | 10.78% | 6.63% | 88.33% |
| DDAM-1 | 15.39% | 2.64% | 2.33% |
| HA | 13.9% | 5.49% | 94.66% |
| NMN | 18.08% | 6.92% | 40.00% |
| Mix | 21.21% | 19.04% | 129.33% |

**Fig.S5** The impact of composite hydrogel on cellular viability.

**Fig.S6** ITC fitting curve diagram of DOMOS and NMN.

**Fig.S7** ITC fitting curve diagram of DDAM-1and NMN.

**Fig.S8** ITC fitting curve diagram of HA and NMN.

**Table S7** Thermodynamic parameters of interactions between DOMOS/DDAM-1/HA and NMN measured by isothermal titration microcalorimetry (ITC).

| NMN | N | KD (M) | ΔH (kJ/mol) | ΔG (kJ/mol) | -TΔS (kJ/mol) |
| --- | --- | --- | --- | --- | --- |
| DOMOS | 7.4e^-2^ | 283e^-6^ | -1000 | -4.84 | 995 |
| DDAM-1 | 5.0e^-2^ | 91.2e^-6^ | -4.46 | -5.51 | -1.05 |
| HA | 1.3e^-2^ | 252e^-6^ | -6.68 | -4.91 | 1.77 |

Average of duplicates for each. n: stoichiometry, KD: affinity level, ΔH: enthalpy, ΔS: entropy, ΔG: free enthalpy, T: temperature.

**Table S8** Molecular Docking Free Energy Results.

| NMN/Polysaccharides | DDAM-1 | DOMOS | HA |
| --- | --- | --- | --- |
| ΔG/kJ·mol^-1^ | -3.0 | -3.4 | -2.8 |

**Fig.S9** The effect of D-gal on cell viability was assessed using the MTS method.

**Fig.S10** Volcano plot of differentially expressed genes between the control group and the model group.

**Fig.S11** Volcano plot of differentially expressed genes between the drug administration group and the model group.

**Fig.S12** Model group VS Control group down gene KEGG enrichment dot plot.

**Fig.S13** Mix group VS Model group up gene KEGG enrichment dot plot.

**Fig S14** Complete full-length Western blot images of all proteins
